# Relationship between educational level and survival of patients with cancer: A multicentre cohort study

**DOI:** 10.1002/cam4.7141

**Published:** 2024-03-28

**Authors:** Xiao‐Yue Liu, Xi Zhang, Guo‐Tian Ruan, Xin Zheng, Yue Chen, Xiao‐Wei Zhang, Tong Liu, Yi‐Zhong Ge, Han‐Ping Shi, INSCOC Group

**Affiliations:** ^1^ Department of Gastrointestinal Surgery Beijing Shijitan Hospital, Capital Medical University Beijing China; ^2^ Department of Clinical Nutrition Beijing Shijitan Hospital, Capital Medical University Beijing China; ^3^ National Clinical Research Center for Geriatric Diseases Xuanwu Hospital, Capital Medical University Beijing China

**Keywords:** educational status, neoplasms, nutritional status, prognosis

## Abstract

**Background:**

Although socioeconomic factors are important determinants of population mortality, the effect of educational level on the survival of patients with cancer in China is unclear. This study aimed to assess whether educational level is associated with the prognosis of patients with cancer and to explore the mediators of this association.

**Methods:**

This multicentre cohort study included 18,251 patients diagnosed with cancer between May 2013 and December 2018. The main parameters measured were overall survival (OS) and all‐cause mortality. The relationship between educational level and all‐cause mortality was assessed using multifactor‐corrected Cox survival analysis. Logistic regression was used to analyze the association between educational level and patient‐generated subjective global assessment (PG‐SGA).

**Results:**

The mean age of the 18,251 participants (men, 9939 [54.4%]) was 57.37 ± 11.66 years. Multifactorial survival analysis showed that patients survived longer with increasing education (university and above vs. elementary school and below; *p* = *p* = <0.001, HR = 0.84, 95% CI: 0.77–0.92), and the differences were statistically significant in different subgroups. The potential impact factors included sex, age, TNM stage, and PG‐SGA score. Logistic regression showed a significant negative association between educational level and the modifiable factor PG‐SGA (secondary vs. primary and below; *p* = 0.004, HR = 0.90, 95% CI: 0.83–0.97; university and above vs. primary and below; *p* < 0.001, HR = 0.79, 95% CI: 0.71–0.88).

**Conclusions:**

Educational level was a significant prognostic factor for patients with cancer, independent of other known prognostic factors. This association was further improved by modifying the nutritional status.

## 1 INTRODUCTION

Cancer is the leading cause of death worldwide, significantly burdens society, and is a major barrier to increased life expectancy.^1^, ^2^, ^3^ The mortality rate of cancer has decreased in recent years with the rise of new tools such as immunotherapy and targeted therapy. However, the latest data from the National Cancer Centre show that China is number one in the world in terms of new cases and deaths, and the overall crude cancer incidence continues to rise, reflecting the heavy burden of cancer in the country. This highlights population aging and growth and changes in the prevalence and distribution of major cancer risk factors, some of which are related to socioeconomic development.

Socioeconomic factors have long been associated with the risk of various cancers.^4^, ^5^, ^6^ Socioeconomic factors are also important indicators of life expectancy in patients with cancer.^7^, ^8^ Educational level is often seen as a proxy for assessing socioeconomic conditions, which are determined early in life. It deeply influences an individual's employment, income, ability to use the health system, and health awareness.^9^ In several foreign studies, the educational level has been recognized as a valid indicator for assessing the prognosis of patients with cancer.^10^, ^11^, ^12^ A 9‐year compulsory education is the basic education system in our country, and the level of education significantly impacts patient health awareness and the occurrence of health events. The educational level can be changed according to an individual's values and the influence of the surrounding environment. However, no scholars in China have studied the predictive value of the educational level on the prognosis of cancer patients.

Understanding the relationship between education and cancer patient survival can inform population health policies. Therefore, this study aimed to explore the association between the educational level of patients with cancer and their prognosis and to determine the mediating factors that influence the correlation.

## 2 METHODS

### 2.1 Study design and participants

This multicentre cohort study investigated the nutritional status and clinical results of Chinese Common Cancer (INSCOC) patients recruited from May 2013 to December 2018 from multiple hospitals in China. This study included 80 hospitals in 16 different provinces in China; some details of the study have been previously described.^13^ In this multicentre study, the inclusion criteria were^1^ pathological diagnosis of cancer,^2^ age ≥ 18 years, and^3^ complete medical information. Patients meeting any of the following criteria were excluded^1^: hospitalization <48 h^2^; presence of severe infectious diseases, immunodeficiency syndromes, or other serious illnesses; and^3^ refusal to sign an informed consent form. Variables with more than 10% missing values and specific variables with missing values were excluded. A total of 18,251 patients were included after excluding those with missing data for the main variables. The detailed process is illustrated in Figure S1. This study complied with the Declaration of Helsinki and was approved by the institutional ethics committees of all participating institutions. All the participants signed an informed consent form (registration number: ChiCTR1800020329).

### 2.2 Data collection

We collected demographic, laboratory, and pathological data from the medical records and databases, including age, sex, comorbidity, smoking status, alcohol consumption, occupation, residence, tumor types, TNM stage, treatment modality, PG‐SGA score, albumin, hemoglobin, neutrophil count, lymphocyte count, platelets, body mass index (BMI) (low, <18.5 kg/m^2^; normal, 18.5–24 kg/m^2^; high, ≥24 kg/m^2^) and the European Organization for Research and Treatment of Cancer Quality of Life Questionnaire (EORTC QLQ‐C30) scores. All patients underwent a complete nutritional assessment, physical fitness test, and quality of life survey within 48 h of admission and were followed up for clinical outcomes 30 days after hospitalization. They also underwent long‐term follow‐up visits to determine patient survival.

Educational level was categorized as a university degree or higher, secondary school (middle or high school), or elementary school or lower. BMI (kg/m^2^) was calculated as weight/height^2^. The TNM classification was based on the eighth edition of the American Joint Committee on Cancer (AJCC) TNM staging system. Laboratory indicators were sent to a laboratory for professional testing according to hospital standards.

### 2.3 Outcome evaluation

Long‐term information was obtained for all patients via regular telephone calls or outpatient follow‐up visits, which are conducted by trained medical personnel to collect participants' basic information, disease conditions, and activity status. The primary outcome event was the overall survival (OS) of patients with cancer. OS was analyzed using the Kaplan–Meier method. Multivariate Cox regression analysis was used to determine independent predictors of OS. Logistic regression was used to analyze the association between educational level and mediators. OS was defined as the time from diagnosis to death, withdrawal from the study, or last follow‐up visit.

### 2.4 Statistical analysis

In the baseline data, continuous variables are expressed as mean (± standard deviation [SD]), and categorical variables are reported as the number of patients (percentages). The chi‐squared test and *t*‐test were used to compare between‐group differences regarding different variables. The Kaplan–Meier curve was used for the overall assessment of educational attainment and survival in cancer patients. The independent prognostic value of educational level in the OS of patients with cancer was analyzed using univariate and multivariate Cox regression analyses. The association between education level and malnutrition status in different subgroups of cancer patients was determined using logistic regression. Further analysis of the prognostic value of educational attainment on patients with cancer was performed using a multifactor adjusted Cox survival analysis model. We also performed a sensitivity analysis. Statistical significance was set at *p* < 0.05. All statistical analyses were performed using R software version 4.2.2 (R Foundation for Statistical Computing, Vienna, Austria).

## 3 RESULTS

### 3.1 Population characteristics

This study included 18,251 patients. The mean age at baseline was 57.37 ± 11.66 years. The study comprised 9939 (54.4%) men; 4312 (23.6%) patients were diagnosed with lung cancer, 3392 (18.6%) with colorectal cancer, and 2533 (13.9%) with breast cancer. We compared the educational levels regarding some common clinical factors and found that men had higher educational levels than women. Smoking and alcohol consumption were significantly higher among patients with elementary school and lower education than among those with higher education. Higher proportions of albumin, hemoglobin, platelets, BMI, TNM stages I and II, nutritional support therapy, and lower PG‐SGA and EORTC QLQ‐C30 scores were found among those with university level and above than among those with lower education. Specific information is listed in Table 1.

**TABLE 1:** Baseline characteristics.

	Overall (*n* = 18, 251)	Education			*p*‐value
		Primary school and below (*n* = 6394)	Middle school (*n* = 9135)	University or above (*n* = 2722)	
Sex (%)					
Male	9939 (54.5)	3170 (49.6)	5254 (57.5)	1515 (55.7)	<0.001
Female	8312 (45.5)	3224 (50.4)	3881 (42.5)	1207 (44.3)	
Age (mean [SD])	57.37 (11.66)	59.69 (10.71)	56.55 (11.31)	54.70 (13.83)	<0.001
Comorbidity (%),	13,751 (75.3)	4881 (76.3)	6893 (75.5)	1977 (72.6)	<0.001
2	3481 (19.1)	1218 (19.0)	1727 (18.9)	536 (19.7)	
≥2	1019 (5.6)	295 (4.6)	515 (5.6)	209 (7.7)	
Smoking (%)					
No	10,763 (59.0)	3784 (59.2)	5161 (56.5)	1818 (66.8)	<0.001
Yes	7488 (41.0)	2610 (40.8)	3974 (43.5)	904 (33.2)	
Drinking (%)					
No	14,769 (80.9)	5207 (81.4)	7295 (79.9)	2267 (83.3)	<0.001
Yes	3482 (19.1)	1187 (18.6)	1840 (20.1)	455 (16.7)	
Tumor type (%)					
Lung cancer (%)	4312 (23.6)	1498 (23.4)	2209 (24.2)	605 (22.2)	<0.001
Colorectal cancer (%)	3392 (18.6)	1099 (17.2)	1702 (18.6)	591 (21.7)	
Breast cancer (%)	2533 (13.9)	713 (11.2)	1323 (14.5)	497 (18.3)	
Others	8014 (43.9)	3084 (48.2)	3901 (42.7)	1029 (37.8)	
Enteral nutrition (%)					
No	16,723 (91.6)	5882 (92.0)	8399 (91.9)	2442 (89.7)	<0.001
Yes	1528 (8.4)	512 (8.0)	736 (8.1)	280 (10.3)	
Parenteral nutrition (%)					
No	15,932 (87.3)	5637 (88.2)	7967 (87.2)	2328 (85.5)	0.002
Yes	2319 (12.7)	757 (11.8)	1168 (12.8)	394 (14.5)	
Tumor stage (%)					
I	2096 (11.5)	617 (9.6)	1095 (12.0)	384 (14.1)	<0.001
II	4076 (22.3)	1390 (21.7)	2067 (22.6)	619 (22.7)	
III	4923 (27.0)	1804 (28.2)	2417 (26.5)	702 (25.8)	
IV	7156 (39.2)	2583 (40.4)	3556 (38.9)	1017 (37.4)	
Surgery (%)					
No	13,258 (72.6)	4601 (72.0)	6644 (72.7)	2013 (74.0)	0.143
Yes	4993 (27.4)	1793 (28.0)	2491 (27.3)	709 (26.0)	
Chemotherapy (%)					
No	8360 (45.8)	2935 (45.9)	4171 (45.7)	1254 (46.1)	0.915
Yes	9891 (54.2)	3459 (54.1)	4964 (54.3)	1468 (53.9)	
Albumin (mean [SD])	39.76 (12.05)	39.19 (10.62)	39.94 (12.81)	40.50 (12.57)	<0.001
Hb (mean [SD])	122.66 (24.44)	120.82 (25.51)	123.34 (23.94)	124.74 (23.21)	<0.001
NLR (mean [SD])	3.51 (3.53)	3.53 (3.37)	3.52 (3.54)	3.47 (3.82)	0.757
PLT (mean [SD])	233.30 (90.02)	236.46 (90.63)	231.63 (90.01)	231.51 (88.46)	0.002
BMI (mean (SD))	22.60 (3.45)	22.51 (3.53)	22.66 (3.42)	22.62 (3.35)	0.029
PGSGA (mean [SD])	5.61 (4.76)	6.01 (4.88)	5.48 (4.70)	5.14 (4.58)	<0.001
EORTC QLQ‐C30 (mean [SD])	48.17 (12.50)	48.44 (13.16)	48.08 (12.08)	47.84 (12.25)	0.070

Abbreviations: BMI, body mass index; EORTCQLQ‐C30, European Organization for Research and Treatment of Cancer Quality of Life Questionnaire; Hb, hemoglobin; NLR, neutrophil‐to‐lymphocyte ratio; PLT, platelet; BMI, body mass index.

### 3.2 Distribution of educational level among patients with different tumor types

We further analyzed the distribution of tumor types in populations with different educational levels. Colorectal and breast cancers were found to increase with increasing educational levels, whereas lung, gastric, esophageal, and nasopharyngeal cancers were more frequent in people with low educational levels (Figure S2).

### 3.3 Survival outcomes according to educational level

**FIGURE 1:**
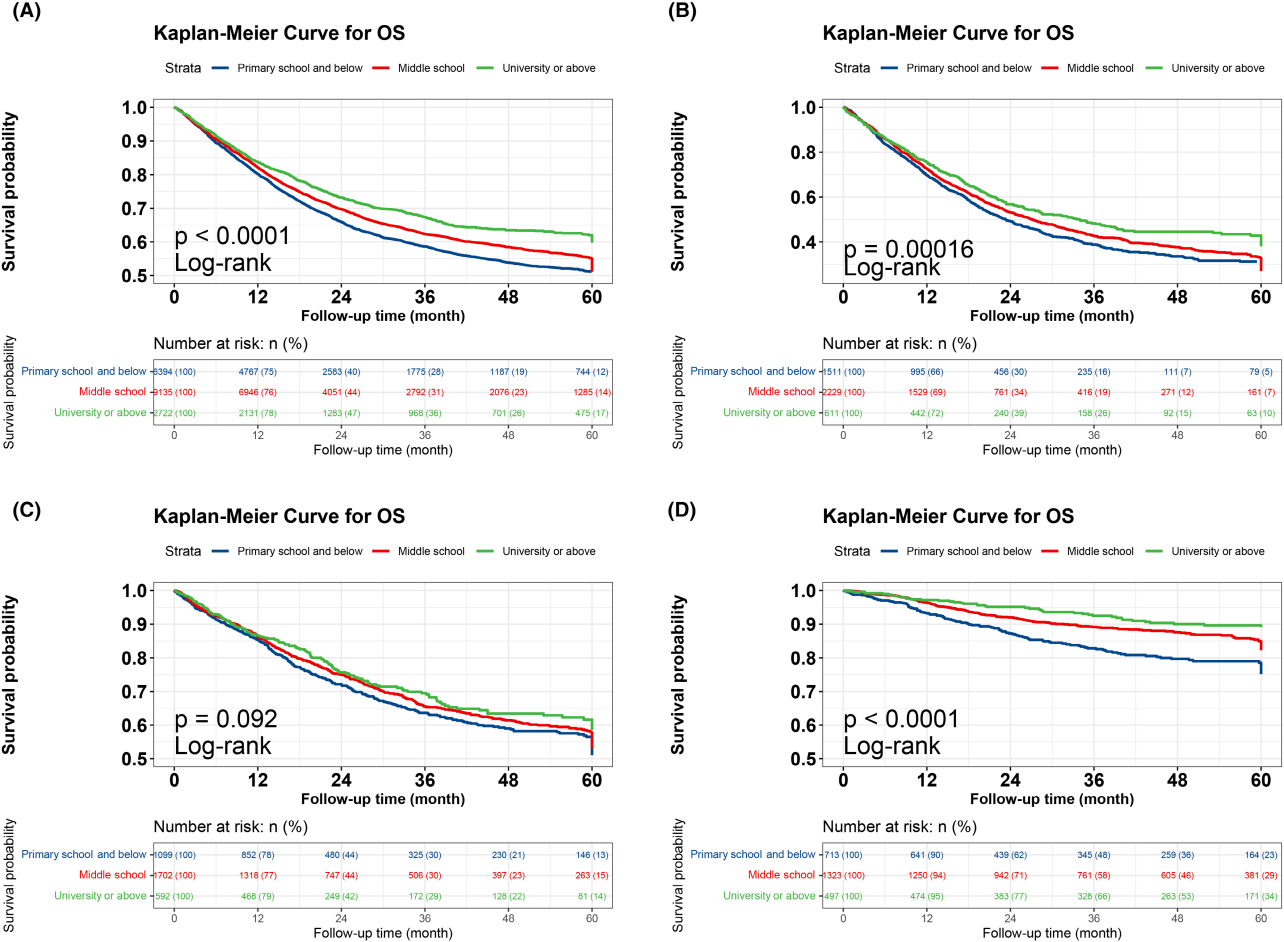
Relationship between education level and prognosis in patients with cancer. (A) K–M curve of education level and overall cancer survival. (B) K–M curve of education level and lung cancer survival. (C) K–M curve of education level and colorectal cancer survival. (D) K–M curve of education level and breast cancer survival.

**FIGURE 2:**
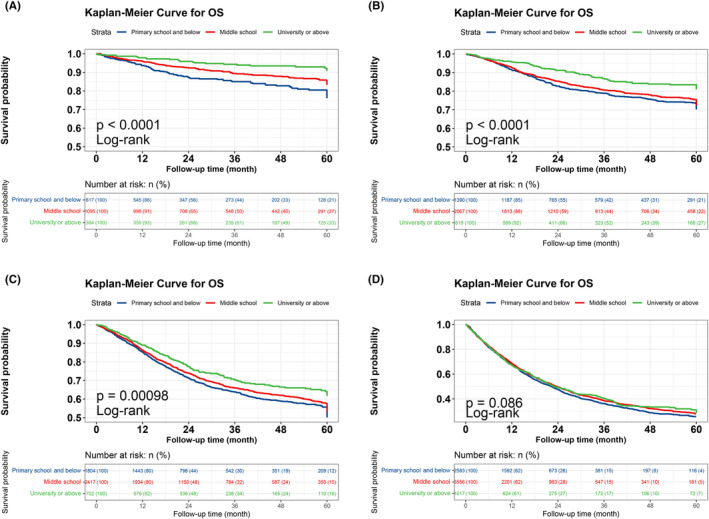
Relationship between education level and prognosis in patients with cancer in different tumor stages. (A) K–M curve of education level and overall cancer survival in stage I. (B) K–M curve of education level and overall cancer survival in stage II. (C) K–M curve of education level and overall cancer survival in stage III. (D) K–M curve of education level and overall cancer survival in stage IV.

The Kaplan–Meier survival curve results showed that the survival time was longer in the group with a high educational level than in the group with a low educational level (*p* < 0.001). This result was consistent in patients with lung, colorectal, and breast cancers and different tumor stages (all *p* < 0.10) (Figures 1 and 2). We performed univariate and multifactorial Cox survival analyses of the clinical parameters. In the univariate survival analysis, age, sex, number of comorbidities, smoking, alcohol consumption, occupation, TNM stage, tumor type, different treatment modalities, PG‐SGA, and BMI were all associated with OS in patients with cancer (all *p* < 0.001). The multivariate survival analysis evaluated clinical parameters (including educational level) that were statistically significant in the univariate survival analysis and found sex, age, number of comorbidities, smoking, TNM stage, treatment modality, PG‐SGA, educational level were effective prognostic factors for patients with cancer (Table S1).

We further investigated the prognostic value of educational level in all patients with cancer and those grouped according to tumor types. We constructed different correction models to reduce clinical bias, and the results showed that in the total population, a higher educational level was associated with a better prognosis in patients with cancer (University and above vs. elementary school and below; crude model: *p* < 0.001, HR = 0.72, 95% CI: 0.67–0.78; model a: *p* < 0.001, HR = 0.79, 95% CI: 0.73–0.86; model b: *p* < 0.001, HR = 0.84, 95% CI: 0.78–0.91; model c: *p* = <0.001, HR = 0.84, 95% CI: 0.77–0.92). Educational level remained an independent prognostic factor for patient survival after different model corrections. Compared with elementary school education or less, university and higher education reduced mortality risk in different types of patients with cancer (Table 2).

**TABLE 2:** Cox proportional analyses of education levels to predict all‐cause mortality for patients with cancer.

	Crude HR (95% CI)	*p*‐value	Adjusted HR (95% CI)^a^	*p*‐value	Adjusted HR (95% CI)^b^	*p*‐value	Adjusted HR (95% CI)^c^	*p*‐value
Overall patients								
Primary school and below	Ref.		Ref.		Ref.		Ref.	
Middle school	0.88 (0.83,0.92)	<0.001	0.93 (0.88, 0.98)	0.005	0.97 (0.92, 1.02)	0.213	0.97 (0.91, 1.02)	0.253
University or above	0.72 (0.67,0.78)	<0.001	0.79 (0.73, 0.86)	<0.001	0.84 (0.78, 0.91)	<0.001	0.84 (0.77, 0.92)	<0.001
Lung cancer								
Primary school and below	Ref.		Ref.		Ref.		Ref.	
Middle school	0.90 (0.82,0.98)	0.018	0.89 (0.81, 0.97)	0.009	0.93 (0.85, 1.02)	0.13	0.96 (0.87, 1.06)	0.397
University or above	0.76 (0.66,0.87)	<0.001	0.75 (0.65, 0.85)	<0.001	0.76 (0.67, 0.87)	<0.001	0.8 (0.69, 0.93)	0.004
Colorectal cancer								
Primary school and below	Ref.		Ref.		Ref.		Ref.	
Middle school	0.91 (0.79,1.04)	0.156	0.95 (0.83, 1.08)	0.426	0.94 (0.82, 1.08)	0.416	0.87 (0.75, 1.02)	0.079
University or above	0.80 (0.67,0.97)	0.021	0.84 (0.69, 1.01)	0.062	0.75 (0.62, 0.9)	0.002	0.63 (0.51, 0.79)	<0.001
Breast cancer								
Primary school and below	Ref.		Ref.		Ref.		Ref.	
Middle school	0.64 (0.50,0.80)	<0.001	0.66 (0.52, 0.84)	0.001	0.83 (0.66, 1.06)	0.135	0.89 (0.68, 1.17)	0.400
University or above	0.41 (0.29,0.58)	<0.001	0.46 (0.32, 0.65)	<0.001	0.75 (0.53, 1.07)	0.108	0.98 (0.64, 1.51)	0.942

Abbreviations: CI, confidence interval; HR, hazard ratio.

^a^ Model a: Adjusted for age, sex, BMI.

^b^ Model b: Adjusted for age, sex, BMI, tumor stage, surgery, chemotherapy, radiotherapy, tumor type.

^c^ Model c: Adjusted for age, sex, BMI, tumor stage, surgery, chemotherapy, radiotherapy, tumor type, comorbidity, smoking, residence, occupation, PGSGA, center.

### 3.4 Sensitivity analysis

Considering the level of education as a prognostic indicator for patients with cancer, we performed a sensitivity analysis after excluding information on patients who died within 6 months. This grouping was consistent with that of a previous study. Sensitivity analysis showed that a high educational level remained an independent prognostic indicator for patients with cancer (Table S2). In addition, we performed subgroup analyses of different socioeconomic factors. Educational level remained an independent prognostic factor for patient survival across residences. Compared with elementary school education or less, university and higher education reduced the risk of death in urban and rural cancer patients. Moreover, among patients with different occupations, higher educational levels showed a protective trend for survival after multifactorial correction; however, this difference was not statistically significant. In the retired population, high educational levels demonstrated a significant protective effect (Table S3).

### 3.5 PG‐SGA as a mediator of the association between educational level and prognosis

We analyzed the mediating effects of common clinical factors such as age, sex, TNM stage, PG‐SGA, tumor type, smoking, and BMI on the correlation between educational level and cancer patient survival. We found a 33.7% correlation with TNM stage, 20% with age, 14.7% with sex, and 11.4% with PG‐SGA (all *p* < 0.001), whereas the remaining factors were less relevant (Figures S3 and S4). We analyzed the PG‐SGA scores of different levels of education and found that elementary school and below exhibited significantly higher scores than higher educational levels, with consistent results across the TNM stages. The PG‐SGA scores were higher with higher TNM stages at all educational levels (Figure S5).

### 3.6 Association between malnutrition and educational level in patients with cancer

The association between educational level and PG‐SGA was explored: a higher educational level was associated with a lower likelihood of malnutrition, and the results remained statistically significant after multifactorial correction (secondary vs. primary and below, model b; *p* = 0.004, HR = 0.90, 95% CI: 0.83–0.97; university and above versus primary and below. model b; *p* < 0.001, HR = 0.79, 95% CI: 0.71–0.88). We further performed a subgroup analysis based on potential confounders and found that results in TNM stages I and II, age < 65 years, female, were consistent with the total population. However, TNM stages III and IV, age ≥ 65 years, and men with higher educational levels showed a protective trend against malnutrition, but the difference was not statistically significant (Table 3).

**TABLE 3:** Logistic regression of the association between education level and PGSGA.

	OR (95% CI)	*p*‐value	OR (95% CI)^a^	*p*‐value	OR (95% CI)^b^	*p*‐value
Total patients						
Primary school and below	Ref.		Ref.		Ref.	
Middle school	0.81 (0.76, 0.86)	<0.001	0.88 (0.82, 0.95)	<0.001	0.90 (0.83, 0.97)	0.004
University or above	0.72 (0.66, 0.79)	<0.001	0.82 (0.75, 0.91)	<0.001	0.79 (0.71, 0.88)	<0.001
TNM I stage						
Primary school and below	Ref.		Ref.		Ref.	
Middle school	0.76 (0.63, 0.93)	0.007	0.81 (0.66, 0.99)	0.042	0.88 (0.70, 1.10)	0.253
University or above	0.56 (0.43, 0.72)	<0.001	0.62 (0.47, 0.81)	<0.001	0.68 (0.50, 0.93)	0.015
TNM II stage						
Primary school and below	Ref.		Ref.		Ref.	
Middle school	0.78 (0.68, 0.90)	<0.001	0.81 (0.70, 0.94)	0.005	0.89 (0.77, 1.05)	0.162
University or above	0.57 (0.47, 0.70)	<0.001	0.64 (0.52, 0.79)	<0.001	0.74 (0.59, 0.93)	0.011
TNM III stage						
Primary school and below	Ref.		Ref.		Ref.	
Middle school	0.87 (0.77, 0.99)	0.033	1.03 (0.91, 1.18)	0.628	0.98 (0.84, 1.13)	0.737
University or above	0.75 (0.63, 0.89)	0.001	0.97 (0.80, 1.17)	0.736	0.83 (0.67, 1.04)	0.101
TNM IV stage						
Primary school and below	Ref.		Ref.		Ref.	
Middle school	0.82 (0.74, 0.91)	<0.001	0.86 (0.77, 0.97)	0.01	0.84 (0.74, 0.94)	0.004
University or above	0.93 (0.80, 1.08)	0.353	0.99 (0.85, 1.16)	0.923	0.85 (0.71, 1.02)	0.077
Age < 65 years						
Primary school and below	Ref.		Ref.		Ref.	
Middle school	0.91 (0.84, 0.98)	0.012	0.92 (0.84, 0.99)	0.033	0.91 (0.83, 0.99)	0.025
University or above	0.76 (0.69, 0.85)	<0.001	0.81 (0.73, 0.91)	<0.001	0.72 (0.63, 0.82)	<0.001
Age ≥ 65 years						
Primary school and below	Ref.		Ref.		Ref.	
Middle school	0.78 (0.69, 0.89)	<0.001	0.82 (0.72, 0.94)	0.004	0.88 (0.76, 1.02)	0.095
University or above	0.81 (0.68, 0.98)	0.029	0.84 (0.69, 1.02)	0.080	0.95 (0.77, 1.18)	0.638
Male						
Primary school and below	Ref.		Ref.		Ref.	
Middle school	0.88 (0.81, 0.96)	0.006	1.02 (0.92, 1.12)	0.734	1.01 (0.91, 1.12)	0.836
University or above	0.85 (0.75, 0.97)	0.013	1.03 (0.91, 1.18)	0.619	0.95 (0.82, 1.10)	0.517
Female						
Primary school and below	Ref.		Ref.		Ref.	
Middle school	0.69 (0.63, 0.76)	<0.001	0.77 (0.70, 0.85)	<0.001	0.80 (0.72, 0.90)	<0.001
University or above	0.56 (0.49, 0.64)	<0.001	0.65 (0.57, 0.75)	<0.001	0.68 (0.57, 0.80)	<0.001

Abbreviations: CI, confidence interval; OR, odds ratio.

^a^ Model a: Adjusted for age, sex, BMI.

^b^ Model b: Adjusted for age, sex, BMI, comorbidity, smoking, drinking, tumor stage, residence, occupation, nutritional supplement, surgery, chemotherapy, radiotherapy.

## 4 DISCUSSION

Cancer is becoming increasingly prevalent in middle‐aged and elderly populations and seriously affects life expectancy. Current studies show that many common social life factors, such as smoking,^14^ alcohol consumption,^15^ and diet,^16^ affect the survival of patients with cancer. However, few studies have examined the relevance of socioeconomic factors to the prognosis of patients with cancer. To the best of our knowledge, this is the first study to examine the prognostic value of educational level in a domestic cancer population and to assess the mediating effect of common clinical factors. This cohort study found that 35% of the patients had elementary school or lower, 50% had secondary school, and 15% had a university or higher education. We also found that the survival time was significantly longer in cancer patients with higher education and that PG‐SGA scores could be a modifiable mediator.^3^, ^17^, ^18^

Our study found that the proportion of patients with university or higher education was the lowest among patients with cancer. The frequency of lung, stomach, nasopharyngeal, and esophageal cancers decreased with higher educational levels, whereas the frequency of breast and colorectal cancers increased with higher educational levels. Breast cancer self‐examination and early detection rates are high among individuals with high educational levels. They have children later and have fewer children.^19^ Moreover, the average time to menopause is later in people with higher levels of education, and longer estrogen stimulation of the breast lineage also increases the risk of breast cancer.^20^, ^21^

More educated people drank more often and in greater quantities, which also played a role.^22^ The occurrence of colorectal cancer is closely related to a patient's diet. In populations with high levels of education, the amount and frequency of red meat intake are elevated to some extent, leading to an increased risk of colorectal cancer.^23^ In addition, we evaluated the mediating effect of common clinical factors on educational levels. The results showed that the TNM stage, age, sex, and PG‐SGA were the mediating factors that contributed the most. TNM stage is currently a clinically accepted index for evaluating tumor progression, and its grading needs to be determined based on postoperative pathology and systemic examination, which are more limited in use.^24^, ^25^, ^26^ Age and sex are natural factors that cannot be changed. As age increases, the incidence of cancer increases, and physical function decreases, leading to a poor patient prognosis and quality of life. Moreover, the difference in hormone levels and living habits of different sexes also subtly affect cancer development.^25^, ^27^ PG‐SGA is a commonly used indicator to evaluate malnutrition in patients, which is a valid independent prognostic indicator for patients with cancer and can be improved by nutritional interventions to improve the patient's nutritional status and, thus, prognosis.^28^, ^29^ This is consistent with our analysis, which suggests that improving malnutrition may reduce the effect of educational level on mortality in patients with cancer.

To our knowledge, this is the first multicentre prospective study in China to analyze socioeconomic factors that predict mortality in patients with cancer and explore their potential mediators. Educational level is a well‐recognized indicator of socioeconomic status, and previous international studies have described the relationship between educational level and mortality.^30^, ^31^ A study based on an Italian population showed the importance of educational level on all‐cause mortality, cardiovascular mortality, malignancies (especially lung cancer), and road accidents (only among men).^32^ A cohort study in the Piedmont region of North Carolina found a significant effect of educational levels on total and effective life expectancy for those aged ≥65 years.^11^ In addition, there are several foreign studies on the association between educational level, cancer incidence, and mortality.^33^ Similar to our findings, another meta‐analysis showed that higher educational levels might be associated with an increased risk of breast cancer.^34^ In Swedish patients with cancer, higher educational levels showed a significant negative association with mortality.^12^ This result was also observed in an Australian cancer population.^35^ Puigpinós et al. also found that men with less education had higher mortality rates from the stomach, oral cavity, pharynx, and esophagus, larynx, and lung cancers. Among women, educational inequalities existed for cervical, liver, and colon cancer.^36^ These findings further validate our results. It has also been found that prognostic factors among patients with cancer stratified by different levels of education have different degrees of impact on mortality, with a significant increase in patients with low education.^37^

We were surprised to find that the number of chronic comorbidities increased with higher levels of education, which is not consistent with normal logic. This may be because people with higher levels of education survive longer, are older, and have a higher intake of meat and non‐healthy diets, all of which lead to an increase in chronic conditions. Considering that patients' pre‐existing health conditions could also potentially influence the association between education level and prognosis, we corrected for comorbidities in our analysis and found more consistent results. The mediating effect of comorbidities on the association between education and prognosis of patients with tumors showed no significant mediating effect. We therefore concluded that the presence of prior chronic diseases in this population did not significantly influence the results.

This study has some strengths in that educational level was utilized as a proxy for socioeconomic factors, and information on patients' educational levels at baseline was collected, thus reducing reverse causality. In addition, this is the largest and one of the few prospective cohort studies in China exploring the relationship between educational level and the prognosis of patients with cancer. Nevertheless, this study has some limitations. Although the mediating effects of common clinical factors on education and patient prognosis were explored in this study, there are other potential influencing factors. In addition, this was a multicentre retrospective study, and there may have been some selection bias. Third, this is an observational study based on a Chinese population. Whether the results can be further extended globally remains to be verified.

## 5 CONCLUSIONS

This study is the first to identify educational level as a potential independent prognostic factor in patients with cancer. Patients with university or higher education had significantly longer survival, and nutritional status accounted for 11.4% of the effect. Although improving the nutritional status of patients alone does not fully address inequality in socioeconomic factors, it may improve patient prognosis to some extent.

## AUTHOR CONTRIBUTIONS

**Xiao‐yue Liu:** Data curation (lead); validation (lead); writing – original draft (lead). **Xi zhang:** Conceptualization (equal). **Guo‐Tian Ruan:** Conceptualization (equal). **Xin Zheng:** Data curation (equal); visualization (equal). **Yue Chen:** Writing – review and editing (equal). **Xiao‐wei Zhang:** Conceptualization (equal). **Tong Liu:** Writing – review and editing (equal). **Yi‐Zhong Ge:** Data curation (equal); visualization (equal). **Han‐ping Shi:** Funding acquisition (lead); resources (lead).

## FUNDING INFORMATION

This study was supported by the National Key Research and Development Program (2022YFC2009600 to Dr. Han‐ping Shi).

## CONFLICT OF INTEREST STATEMENT

The authors declare no conflicts of interest.

## ETHICS STATEMENT

This study was conducted according to the guidelines of the Declaration of Helsinki, and all procedures involving research participants were approved by the ethics committee at Beijing Shijitan Hospital, Capital Medical University, Beijing, China. Written informed consent was obtained from all the patients.

## Supporting information


Data S1:

## Data Availability

The datasets used and/or analyzed in the current study are available from the corresponding author upon reasonable request.
