# Supplementary material for: Relationship between educational level and survival of patients with cancer: A multicentre cohort study
Source: Cancer Med. 2024 Mar 28;13(7):e7141. doi: 10.1002/cam4.7141 (PMC10974719; doi:10.1002/cam4.7141)

Supplementary Table 1. Univariate and multivariate survival analysis between common clinical factors and patients with cancer.

| Characteristics |  | Uni_HR(95%CI) | *P*-value | Mul_HR(95%CI) | *P*-value |
| --- | --- | --- | --- | --- | --- |
| Sex | Male | ref. |  |  |  |
|  | Female | 0.55 (0.53,0.58) | <0.001 | 0.85 (0.80 , 0.91) | <0.001 |
| Age | <65 | ref. |  |  |  |
|  | ≥65 | 1.70 (1.62,1.79) | <0.001 | 1.24 (1.17 , 1.31) | <0.001 |
| Comorbidity | 0 | ref. |  |  |  |
|  | 1 | 1.17 (1.1,1.24) | <0.001 | 1.07 (1.01 , 1.14) | 0.028 |
|  | ≥2 | 1.31 (1.19,1.45) | <0.001 | 1.11 (1.01 , 1.24) | 0.039 |
| Smoking | No | ref. |  |  |  |
|  | Yes | 1.66 (1.58,1.75) | <0.001 | 1.12 (1.05 , 1.19) | 0.001 |
| Drinking | No | ref. |  |  |  |
|  | Yes | 1.40 (1.32,1.48) | <0.001 | 1.05 (0.99 , 1.13) | 0.109 |
| Residence | Rural | ref. |  |  |  |
|  | Urban | 1.00 (0.95,1.05) | 0.96 |  |  |
| Education | Primary school and below | ref. |  |  |  |
|  | Middle school | 0.88 (0.83,0.92) | <0.001 | 0.95 (0.89 , 1.00) | 0.057 |
|  | University or above | 0.72 (0.67,0.78) | <0.001 | 0.83 (0.76 , 0.91) | <0.001 |
| Occupation | Brainwork | ref. |  |  |  |
|  | Physicallabour | 1.37 (1.28,1.47) | <0.001 | 1.03 (0.96 , 1.12) | 0.4 |
|  | Retirement and other | 1.43 (1.33,1.53) | <0.001 | 1.08 (1 , 1.16) | 0.038 |
| Tumor Stage | I | ref. |  |  |  |
|  | II | 1.74 (1.51,2.02) | <0.001 | 1.72 (1.49 , 1.99) | <0.001 |
|  | III | 3.42 (2.99,3.92) | <0.001 | 3.02 (2.63 , 3.47) | <0.001 |
|  | IV | 8.24 (7.23,9.39) | <0.001 | 6.31 (5.52 , 7.23) | <0.001 |
| Surgery | No | ref. |  |  |  |
|  | Yes | 0.52 (0.49,0.56) | <0.001 | 0.77 (0.71 , 0.83) | <0.001 |
| Chemotherapy | No | ref. |  |  |  |
|  | Yes | 1.13 (1.08,1.19) | <0.001 | 0.85 (0.81 , 0.91) | <0.001 |
| Radiotherapy | No | ref. |  |  |  |
|  | Yes | 0.77 (0.71,0.84) | <0.001 | 0.63 (0.57 , 0.68) | <0.001 |
| Tumor type |  |  |  |  |  |
| Lung cancer |  | ref. |  |  |  |
| Colorectal cancer |  | 0.49 (0.46,0.53) | <0.001 | 0.56 (0.52 , 0.61) | <0.001 |
| Breast cancer |  | 0.15 (0.14,0.17) | <0.001 | 0.35 (0.31 , 0.39) | <0.001 |
| Others |  | 0.59 (0.56,0.62) | <0.001 | 0.71 (0.67 , 0.76) | <0.001 |
| PGSGA | <4 | ref. |  |  |  |
|  | ≥4 | 2.13 (2.02,2.24) | <0.001 | 1.65 (1.56 , 1.74) | <0.001 |
| BMI | <18.5 | ref. |  |  |  |
|  | 18.5-23.9 | 0.6 (0.56,0.64) | <0.001 | 0.76 (0.70 , 0.81) | <0.001 |
|  | ≥24 | 0.45 (0.42,0.49) | <0.001 | 0.68 (0.63 , 0.74) | <0.001 |

Supplementary Table 2. Cox proportional analysis of education level predicted all-cause mortality of cancer patients excluding death within 6 months.

|  | **crude HR(95%CI)** | ***P*-value** | **adjusted HR (95% CI)^a^** | ***P*-value** | **adjusted HR(95%CI)^b^** | ***P*-value** | **adjusted HR(95%CI)c** | ***P*-value** |
| --- | --- | --- | --- | --- | --- | --- | --- | --- |
| **Education** |  |  |  |  |  |  |  |  |
| **Primary school and below** | ref. |  | ref. |  | ref. |  | ref. |  |
| **Middle school** | 0.87 (0.82,0.93) | <0.001 | 0.91 (0.86 , 0.97) | 0.005 | 0.96 (0.9 , 1.02) | 0.226 | 0.99 (0.92 , 1.06) | 0.682 |
| **University or above** | 0.7 (0.64,0.77) | <0.001 | 0.76 (0.7 , 0.84) | <0.001 | 0.82 (0.75 , 0.9) | <0.001 | 0.86 (0.77 , 0.96) | 0.005 |

Model a: Adjusted for age, sex, BMI; Model b: Adjusted for age, sex, BMI, tumor stage, surgery, chemotherapy, radiotherapy, tumor type; Model c: Adjusted for age, sex, BMI, tumor stage, surgery, chemotherapy, radiotherapy, tumor type, comorbidity, smoking, residence, occupation, PGSGA. Abbreviations: HR, hazard ratio; CI, confidence interval.

Supplementary Table 3. Cox proportional analysis of education level predicted all-cause mortality of cancer patients according to socioeconomic factors.

|  | **crude HR(95%CI)** | P-value | adjusted HR (95% CI)^a^ | P-value | adjusted HR(95%CI)^b^ | P-value | **adjusted HR(95%CI)c** | P-value |
| --- | --- | --- | --- | --- | --- | --- | --- | --- |
| **By residence** |  |  |  |  |  |  |  |  |
| **Urban** |  |  |  |  |  |  |  |  |
| **Primary school and below** | ref. |  | ref. |  | ref. |  | ref. |  |
| **Middle school** | 0.84 (0.78,0.9) | <0.001 | 0.89 (0.83 , 0.96) | 0.003 | 0.97 (0.9 , 1.04) | 0.352 | 0.99 (0.91 , 1.07) | 0.756 |
| **University or above** | 0.69 (0.63,0.76) | <0.001 | 0.76 (0.69 , 0.83) | <0.001 | 0.83 (0.76 , 0.92) | <0.001 | 0.89 (0.8 , 0.98) | 0.022 |
| **Rural** |  |  |  |  |  |  |  |  |
| **Primary school and below** | ref. |  | ref. |  | ref. |  | ref. |  |
| **Middle school** | 0.88 (0.81,0.96) | 0.003 | 0.95 (0.87 , 1.03) | 0.214 | 0.96 (0.89 , 1.05) | 0.412 | 0.97 (0.89 , 1.06) | 0.541 |
| **University or above** | 0.68 (0.53,0.86) | 0.001 | 0.81 (0.63 , 1.03) | 0.079 | 0.82 (0.65 , 1.05) | 0.116 | 0.8 (0.62 , 1.03) | 0.086 |
| **P for interaction** | 0.726 | | | | | | | |
| **By occupation** |  |  |  |  |  |  |  |  |
| **Brainwork** |  |  |  |  |  |  |  |  |
| **Primary school and below** | ref. |  | ref. |  | ref. |  | ref. |  |
| **Middle school** | 0.97 (0.8,1.17) | 0.744 | 1.01 (0.84 , 1.23) | 0.888 | 1.06 (0.87 , 1.28) | 0.58 | 1.08 (0.89 , 1.31) | 0.444 |
| **University or above** | 0.76 (0.62,0.93) | 0.007 | 0.82 (0.67 , 1) | 0.055 | 0.87 (0.71 , 1.06) | 0.174 | 0.93 (0.75 , 1.16) | 0.52 |
| **Physical labour** |  |  |  |  |  |  |  |  |
| **Primary school and below** | ref. |  | ref. |  | ref. |  | ref. |  |
| **Middle school** | 0.95 (0.87,1.03) | 0.214 | 1.01 (0.93 , 1.1) | 0.778 | 1.01 (0.92 , 1.1) | 0.853 | 1.02 (0.93 , 1.12) | 0.653 |
| **University or above** | 0.62 (0.4,0.96) | 0.031 | 0.78 (0.5 , 1.22) | 0.273 | 0.84 (0.54 , 1.31) | 0.438 | 0.86 (0.55 , 1.34) | 0.493 |
| **Retirement and other** |  |  |  |  |  |  |  |  |
| **Primary school and below** | ref. |  | ref. |  | ref. |  | ref. |  |
| **Middle school** | 0.81 (0.75,0.88) | <0.001 | 0.86 (0.79 , 0.93) | <0.001 | 0.92 (0.85 , 1) | 0.058 | 0.93 (0.85 , 1.01) | 0.072 |
| **University or above** | 0.82 (0.73,0.92) | 0.001 | 0.79 (0.7 , 0.88) | <0.001 | 0.84 (0.74 , 0.94) | 0.002 | 0.84 (0.75 , 0.95) | 0.004 |
| **P for interaction** | 0.206 | | | | | | | |

Model a: Adjusted for age, sex, BMI; Model b: Adjusted for age, sex, BMI, tumor stage, surgery, chemotherapy, radiotherapy; Model c: Adjusted for age, sex, BMI, tumor stage, surgery, chemotherapy, radiotherapy, comorbidity, smoking, residence, occupation, PGSGA. Abbreviations: HR, hazard ratio; CI, confidence interval.

Supplementary Figure 1. Flow chart.





Supplementary Figure 2. Education level of patients with different tumor types.


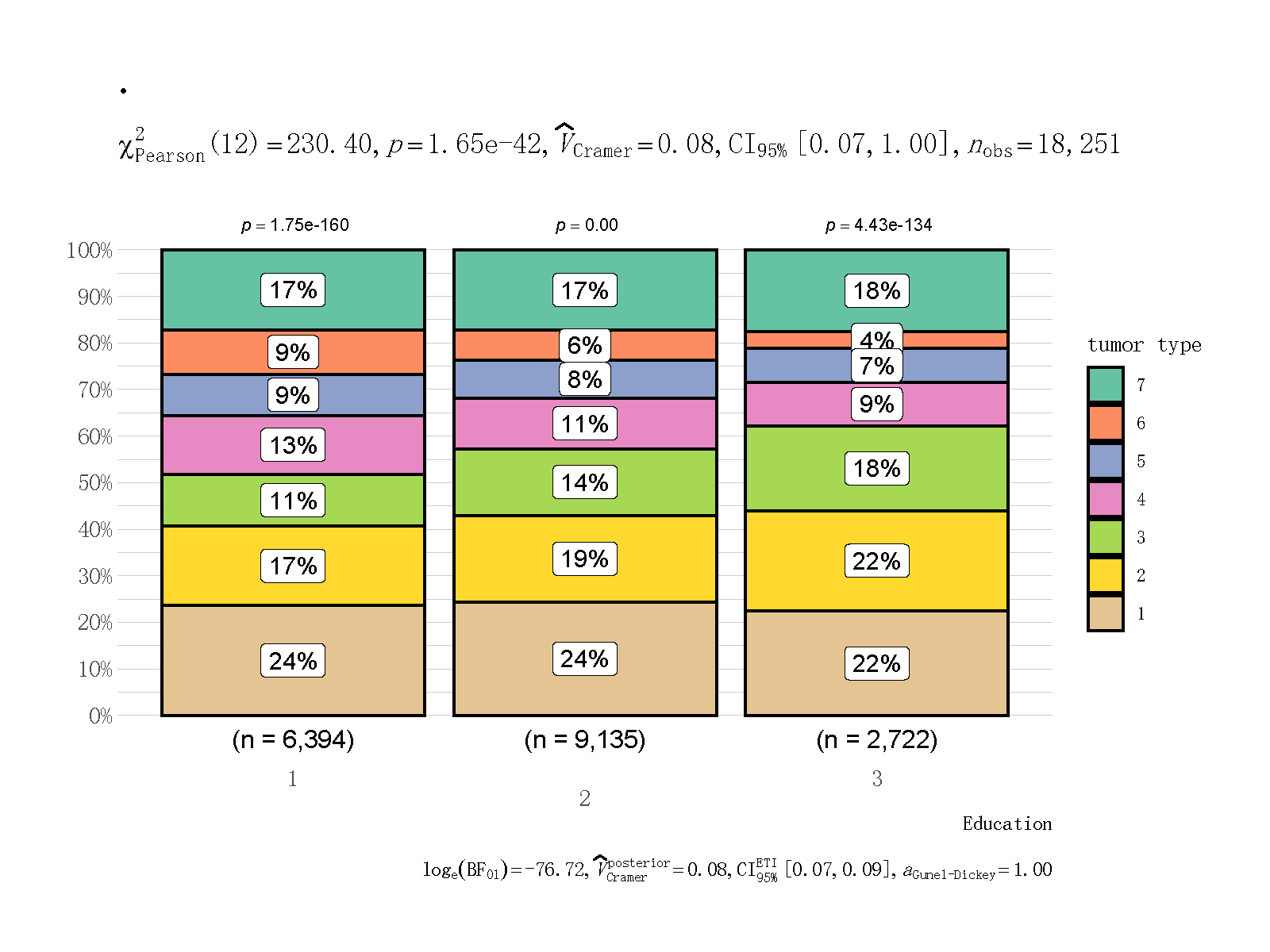


Tumor type: 1, lung cancer; 2, colorectal cancer; 3, breast cancer; 4. gastric cancer; 5, nasopharynx cancer; 6, esophagus cancer; 7, other types.

Education: 1. Primary school and below; 2, Middle school; 3, University or above.

Supplementary Figure 3. Mediating effect of different indicators on education level and prognosis of cancer patients


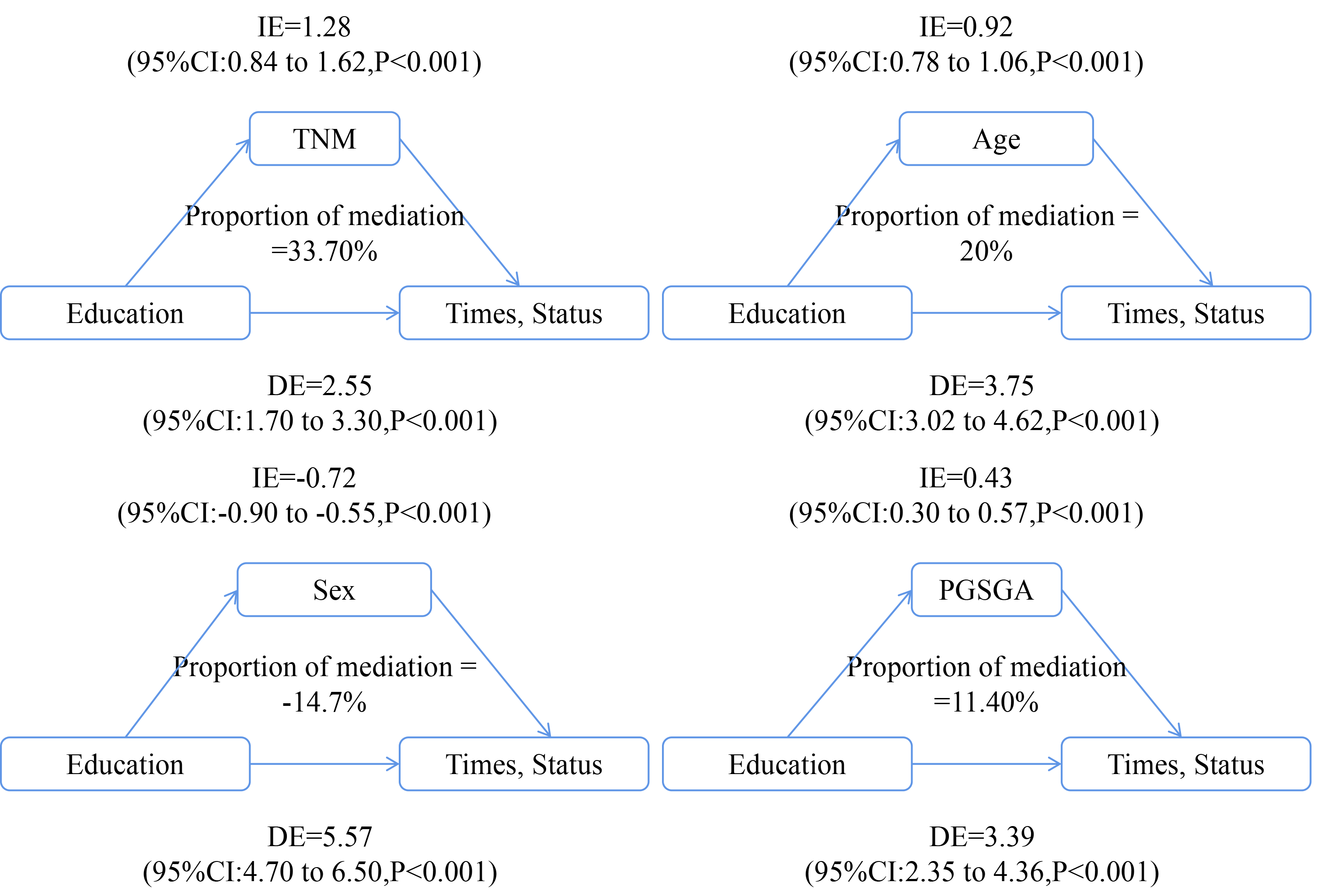


Supplementary Figure 4. Mediating effect of different indicators on education level and prognosis of cancer patients


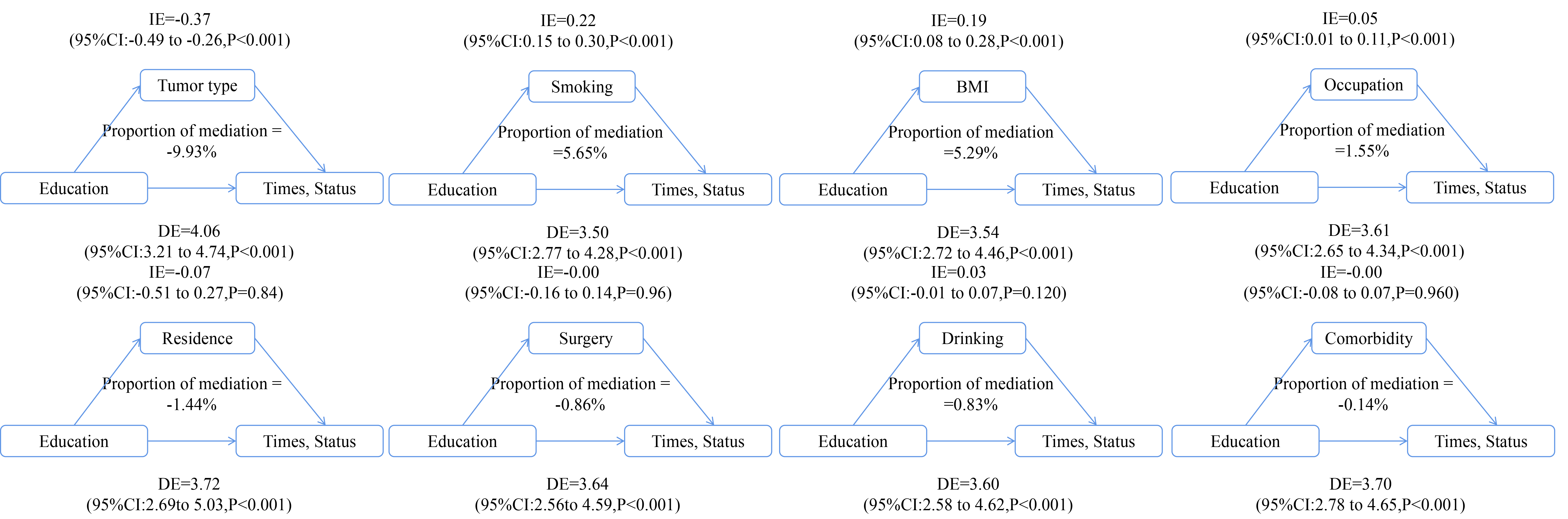


Supplementary Figure 5. The distribution of PGSGA in patients with different education levels.


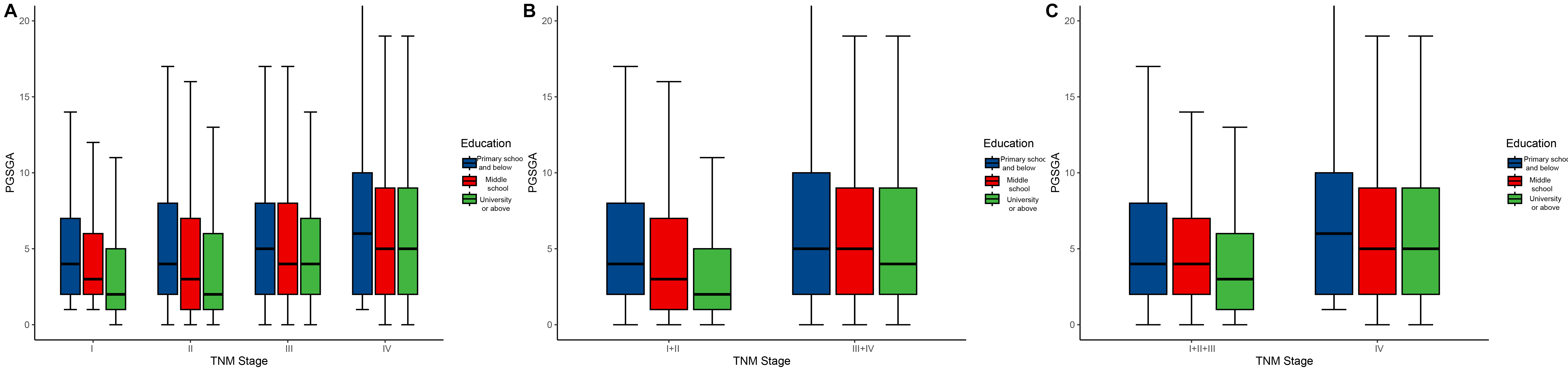

Supplement: Supplementary file 1 — Data S1: [file CAM4-13-e7141-s001.doc]
